# Prospective Trial to Compare Direct and Indirect Laryngoscopy Using C-MAC PM® with Macintosh Blade and D-Blade® in a Simulated Difficult Airway

**DOI:** 10.1155/2019/1067473

**Published:** 2019-04-01

**Authors:** Florian Jürgen Raimann, Philipp Edmund Dietze, Colleen Elizabeth Cuca, Dirk Meininger, Paul Kessler, Christian Byhahn, Daniel Gill-Schuster, Kai Zacharowski, Haitham Mutlak

**Affiliations:** ^1^Department of Anesthesiology, Intensive Care Medicine and Pain Therapy, University Hospital Frankfurt, Theodor-Stern Kai 7, 60590 Frankfurt, Germany; ^2^Main-Kinzig-Clinic, Department of Anesthesia, Intensive Care Medicine and Pain Therapy, Herzbachweg 14, 63571 Gelnhausen, Germany; ^3^Clinic of Anesthesiology, Intensive Care and Pain Therapy, Orthopedic Clinic Friedrichsheim, Marienburgstraße 2, 60528 Frankfurt am Main, Germany; ^4^Evangelical Hospital Oldenburg, Department of Anesthesia, Intensive Care Medicine and Pain Therapy, Steinweg 13-17, 26122 Oldenburg, Germany

## Abstract

*Objective*. Evaluation of C-MAC PM® in combination with a standard Macintosh blade size 3 in direct and indirect laryngoscopy and D-Blade® in indirect laryngoscopy in a simulated difficult airway. Primary outcome was defined as the best view of the glottic structures. Secondary endpoints were subjective evaluation and assessment of the intubation process. *Methods*. Prospective monocentric, observational study on 48 adult patients without predictors for difficult laryngoscopy/tracheal intubation undergoing orthopedic surgery. Every participant preoperatively received a cervical collar to simulate a difficult airway. Direct and indirect laryngoscopy w/o the BURP maneuver with a standard Macintosh blade and indirect laryngoscopy w/o the BURP maneuver using D-Blade® were performed to evaluate if blade geometry and the BURP maneuver improve the glottic view as measured by the Cormack-Lehane score.* Results*. Using a C-MAC PM® laryngoscope, D-Blade® yielded improved glottic views compared with the Macintosh blade used with either the direct or indirect technique. Changing from direct laryngoscopy using a Macintosh blade to indirect videolaryngoscopy using C-MAC PM® with D-Blade® improved the Cormack-Lehane score from IIb, III, or IV to I or II in 31 cases. *Conclusion*. The combination of C-MAC PM® and D-Blade® significantly enhances the view of the glottis compared to direct laryngoscopy with a Macintosh blade in patients with a simulated difficult airway. *Trial Registration Number*. This trial is registered under number NCT03403946.

## 1. Introduction

Patients with an unexpectedly difficult airway requiring endotracheal intubation (ETI) remain extremely challenging for emergency physicians, and intubation failure with subsequent hypoxic complications still represents the majority of cases in a closed claim analysis [1].

The incidence of major complications in airway management of 1 in 5,500 was estimated in the Fourth National Audit Project in the UK [2].

Particularly in the prehospital setting, an increased incidence of a difficult airway up to 14.8% is described [3]. Cervical spine immobilization in trauma patients with a collar is common. Cervical collars lead to a reduced reclination of the head and in a reduced interincisor distance. This can make direct visualization of the glottis challenging and the incidence of a difficult airway increases up to 64% [4].

In the last decade, the use of videolaryngoscopes for endotracheal intubation has become routine in the clinical setting, especially in (unexpected) difficult airway management [5–7]. Videolaryngoscopy (VL) eliminates the need for a direct line of sight between the operator and glottis. The potential benefit of VL in difficult airway management is highlighted in different international guidelines [8, 9] and is the subject of a Cochrane analysis [10].

Many different types of VL have been developed in the last years [11] and their application in the clinical setting has been published [12, 13]. In particular, hyperangulated blades have been developed for visibility improvement, although good visibility is not automatically associated with an easy intubation process [14]. For example, D-Blade® with its hyperangulated tip allows a good visualization of the glottis structures in patients with normal and difficult airways [13]. Therefore, only indirect laryngoscopy is possible, requiring an external monitor like C-MAC® or C-MAC PM® [15, 16]. One benefit of the C-MAC PM**®** system is its compact design. By plugging the monitor directly onto the handle, no additional cables or external power supply is required. This has potential advantages especially in the prehospital setting.

We hypothesize that using C-MAC PM® in combination with D-Blade® improves the view of glottic structures in patients with a simulated difficult airway. Our aim was to compare intubation conditions regarding the modified Cormack-Lehane score (CL) [17] between D-Blade® in indirect laryngoscopy or a Macintosh blade in direct and indirect laryngoscopy with C-MAC PM® in a simulated setting of a difficult airway in human subjects. To obtain optimal comparability of the visualization three laryngoscopies with different approaches were performed in one patient.

## 2. Materials and Methods

### 2.1. Study Design

This prospective, single-center study was conducted at the University Hospital Frankfurt, Germany. After approval of the study protocol by the Institutional Ethical Review Board (reference number: E 126/11) the study was carried out in accordance with the Declaration of Helsinki. This study is registered at clinicaltrials.gov (NCT03403946).

### 2.2. Population

Patients requiring general anesthesia for orthopedic surgery were included. Written informed consent was obtained from all participants.

Patients aged <18 or >80 years with a known or expected difficult airway, undergoing urgent or emergent surgery, nonfasted, of American Society of Anesthesiology (ASA) Classes IV-VI, or without consent to participation were excluded from the study.

Preoperative airway evaluation was carried out by assessing the Mallampati score, thyromental distance, cervical spine clearance, and interincisor distance. Difficulty was defined as a Mallampati score III or IV, thyromental distance of <6.5 cm, reclination of <30°, an interincisor distance of <3 cm, presence of a full beard, toothlessness, or a known obstructive sleep apnea syndrome.

### 2.3. Setting

In the operating room, routine monitoring was applied (noninvasive blood pressure, heart rate, and pulse oximetry). Prior to the induction of anesthesia, all patients received a size-adapted cervical collar (Stifneck-Regular®, Laerdal Medical GmbH, Puchheim, Germany) fitted according to the manufacturer's instructions. Interincisor distance, cervical reclination, and the Mallampati score were obtained after collar placement (Table 2).

The cervical collar was then removed, and the patient was then preoxygenated (FiO_2_ = 1.0) for three minutes. Induction of anesthesia was performed intravenously with 2 mg/kg propofol (Fresenius Kabi, Bad Homburg, Germany) and 2 *μ*g/kg fentanyl (Rotexmedica, Luitré, France) in all patients. Neuromuscular blockade was achieved with intravenous 0.6 mg/kg rocuronium (Inresa, Freiburg, Germany). After two to three minutes and absence of spontaneous breathing, the cervical collar was placed again, and laryngoscopy was performed in all participants in the following manner (Figure 2, supplementary online material).

**Figure 1:**
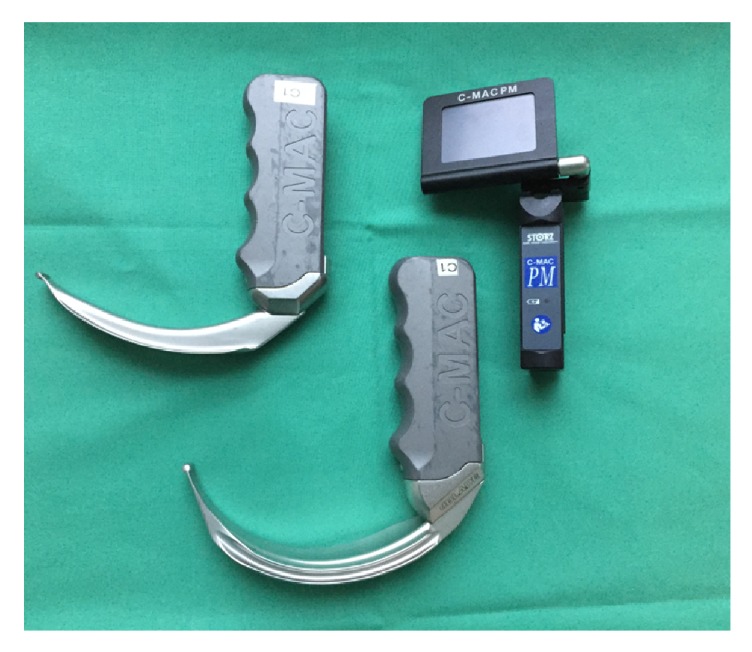
*C-MAC PM®*. C-MAC PM® videolaryngoscope (right), D-Blade® (middle), and Macintosh blade size 3 (left).

First, direct laryngoscopy was performed using a Macintosh blade size 3 with an attached C-MAC PM® monitor (Karl Storz GmbH & Co. KG, Tuttlingen, Germany) with and without applying external laryngeal pressure (the BURP (Backward, Upward, Rightward Pressure) maneuver). To perform direct laryngoscopy, the attached monitor was flipped over. In a second step, view on the monitor for indirect laryngoscopy was allowed and evaluation was performed with and without BURP using the same blade size (Figure 1). The same procedure for indirect laryngoscopy was repeated using an adult D-Blade® (Karl Storz GmbH & Co. KG, Tuttlingen, Germany) and the patient's trachea was finally intubated. We used a size 7.0 tube to intubate the trachea of female patients and a size 8.0 one to intubate the trachea of male patients. The allowed time for the entire examination was limited to 120 seconds. In the case of desaturation (SpO_2_ < 92%), the examination was interrupted and reoxygenation with bag-mask ventilation was performed until SpO_2_ ≥ 98% was achieved. In case of insufficient bag-mask ventilation, the cervical collar was removed. After reoxygenation, the collar was placed again, and the procedure was continued.

Visualization of glottis structures was dichotomized into “good” and “poor” according to the modified CL classification. CL scores IIb, III, and IV were defined as “poor” visualization whereas CL scores I and IIa were defined as “good” visualization. A change of CL score in the same subject, when switching the device or method, was defined as a clinically relevant improvement in visualization (Table 3).

ETI was defined as failed if intubation was not successful within 120 seconds or after two failed tracheal intubation attempts. When ETI failed, the cervical collar was removed, and intubation was performed with the blade of choice. All participating anesthesiologists were board certified with at least five years of clinical experience.

### 2.4. Sample Size Calculation

The calculated sample size to achieve 80% power and to detect a 36% reduction in the grade of glottic view considered as “good visualization” (CL scores I, IIa) was 39 patients.

### 2.5. Statistical Analysis

Statistical analysis was performed using SigmaPlot 12 (Systat Software GmbH, Erkrath, Germany). Calculation of significance was carried out by using Wilcoxon's signed rank test, the paired* t*-test, and Fisher's exact test. For multiple comparisons, one-way ANOVA and the Bonferroni post hoc tests were used. Level of significance was assumed with a probability of type I error of less than 5% (*p*<0.05). The Hodges-Lehmann estimator was used for evaluation of median differences. Values were expressed as number (percent), mean ± SD, or median (IQR), as appropriate.

### 2.6. Endpoints

The primary outcome parameter was the best view of the glottic structures according to the modified Cormack-Lehane (CL) scoring system by Yentis and Lee (Figure 3).

To detect the effect of changing devices or technique dichotomous data was calculated (Table 3).

As a secondary endpoint, all investigators were asked to subjectively evaluate the process of ETI with D-Blade®: Grade A: uncomplicated ETI with guide rod, Grade B: challenging ETI, readjustment or usage of BURP necessary, Grade C: ETI using a stylet, and Grade D: failed ETI (Table 4).

Finally, all investigators were asked for their subjective assessment from 0 = dissatisfied to 100 = fully satisfied when using D-Blade®.

## 3. Results

Statistical analysis (the Shapiro-Wilk Test) confirmed that the data was not normally distributed. Therefore, all data were presented as medians and IQR.

**Figure 2:**
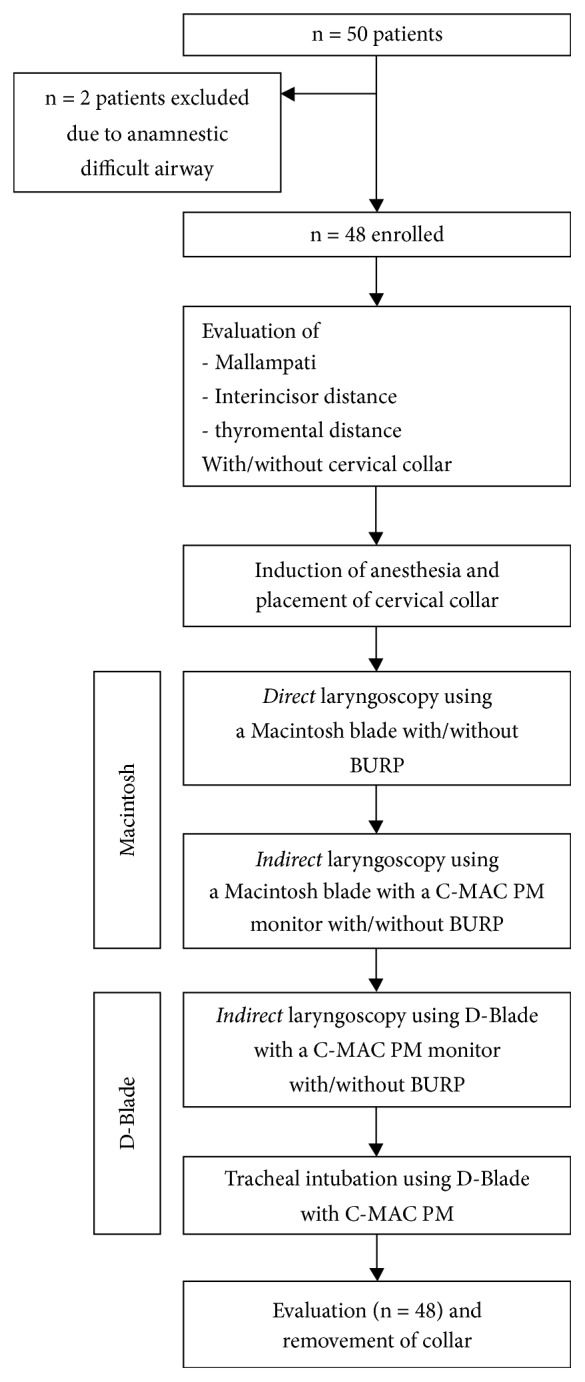
*Study Flowchart*. Flowchart study design. Supplementary online material.

Fifty patients were screened for study inclusion. Two patients were excluded for not fulfilling the inclusion criteria due to known previous intubation difficulties. 48 patients were examined (24 male, 24 female), and in all cases the complete protocol was fulfilled (Figure 2, supplementary online material). Demographic data are depicted in Table 1.

When the cervical collar was placed, the Mallampati score, interincisor distance, and reclination significantly deteriorated, creating a scenario with difficult ETI conditions (Table 2).

### 3.1. Primary Endpoint: Visualization of Glottis Structure

**Figure 3:**
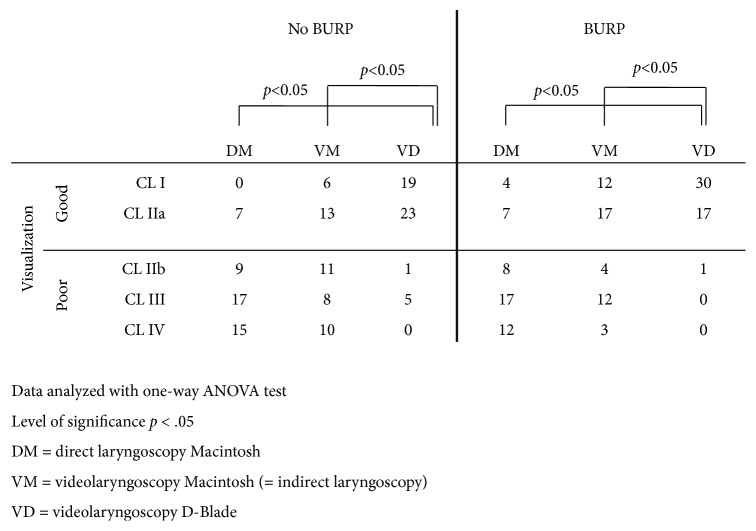
Results of examination of glottis view using the Cormack-Lehane score.

The visualization of the glottis is presented in Figure 3.

The worst visualization was obtained by direct laryngoscopy with statistically significant improvement (*p* <0.05) when performing indirect laryngoscopy with the C-MAC PM® Macintosh blade and C-MAC PM® D-Blade® (Figure 3). Overall optimization of the visualization was obtained by applying BURP. Again, with direct laryngoscopy the poorest visualization was obtained. Significant improvement was achieved by indirect laryngoscopy either by the C-MAC PM**®** Macintosh blade or by C-MAC PM® D-Blade® (Figure 3).

Converting poor into good visualization was achieved when switching from direct to indirect visualization. Most conversions to good visualization (n=31) were achieved when indirect laryngoscopy with D-Blade® and BURP was performed after direct laryngoscopy with a Macintosh blade in the simulated difficult airway. Since only changes based on Cormack-Lehane grades 3 and 4 were examined and not changes based on grades 2a, 2b, and 1, Table 3 shows the different numbers of evaluated patients.

### 3.2. Secondary Endpoint: Subjective Satisfaction

All investigators were asked to subjectively evaluate the ETI process by predefined categories and the results are displayed in Table 4.

Intubation according to the protocol was performed with D-Blade® using the indirect technique. In 45%, the intubation process was rated “easy”; in 20% (n=10), intubation failed despite good visualization (at least CL score IIa). After removing the cervical collar in these patients, ETI was successful.

Subjective satisfaction by the laryngoscopists was determined by a numeric rating scale (0 = dissatisfied to 100 = fully satisfied). Overall, in 48 ETI instances an average of 55.2 was reached (median = 70, IQR: 30/80). No intervention had to be interrupted due to desaturation (SpO_2_ < 92%).

## 4. Discussion

VL has the potential to enhance the glottic view in patients with difficult airway management. Previous studies showed faster visualization [18] and better visibility of the glottis structures when using indirect laryngoscopy in a normal and simulated difficult airway [12, 19]. A limitation in previous studies was that the results regarding visualization and intubation success were made on the basis of interindividual comparison and not performed in one patient. Therefore, we investigated the effect of using the C-MAC PM® system with D-Blade® for indirect laryngoscopy compared to a Macintosh blade for direct and indirect laryngoscopy in patients with simulated difficult airways in terms of visualization of the glottis structures according to the modified CL score [17].

After simulating a difficult airway by the placement of a cervical collar, the predictors for difficult intubation deteriorated congruent to studies by Byhahn [12] and Yang [20], indicating a successfully manufactured difficult airway related by the reduction of movement in the atlantooccipital joint and the prevention of an improved Jackson position/sniffing position. This led to more difficult intubation conditions and can be explained by the fact that an optimization of the oral and pharyngeal axis is no longer possible [21].

The best visualization of the glottic structures was obtained by indirect laryngoscopy with D-Blade®. However, in ten cases ETI was unsuccessful despite good visualization (average CL score IIa). A possible explanation may be the hyperangulation of D-Blade®, which provides a good view but makes it difficult to advance the tube through the glottis [14, 22]. This has been similarly described for hyperangulated blades by other manufacturers. Wallace et al. showed that direct laryngoscopy with McGrath™ MAC resulted in poorer visualization in comparison to indirect visualization [23]. Although the blade geometry of McGrath™ MAC differs from C-MAC PM® D-Blade®, the results are comparable in terms of the influence of angulated blades. The C-MAC Macintosh blade differs in terms of angulation slightly from the classic Macintosh blade and therefore offers the possibility of using the device directly and indirectly.

Beside the choice of the used VL an important factor in successful intubation is the level of experience with the used device. Lye et al. demonstrated that successful intubation correlates with the experience and regular usage [24]. A contributing factor may be eye-arm coordination of the user, especially when using angulated blades, and Bakshi et al. demonstrated that novices in using videolaryngoscopes had more unsuccessful ETI cases than experienced and trained professionals [25].

It is difficult to make a general statement about how much training is needed to be competent in the use of a videolaryngoscope. Different learning curves and diverging manual dexterity make a didactic statement difficult. However, training has been shown to improve the success rate [26].

Thus, in addition to regular training, it was suggested that VL should be routinely available for emergencies. VLs are often part of standard operating procedures (SOPs) for managing expected and unexpected difficult airways and clinicians should be capable of using them effectively. Therefore, VL should be used as often as possible to improve experience with the devices [27]. We were able to show a better view of the glottis in a simulated difficult airway by using indirect VL. Changing from direct to indirect laryngoscopy using a Macintosh blade plus the BURP maneuver resulted in improved intubation conditions in 14 patients. Likewise, we found improved intubation conditions in 31 patients when changing from Macintosh to D-Blade® and direct to indirect laryngoscopy and upon applying BURP. These results cover the findings of Serocki et al., who also found better laryngeal exposure and CL scores using VL compared with direct laryngoscopy [16]. However, caution is advised when using the BURP maneuver. Within the scope of cervical spine injuries, this maneuver may cause further damage to already injured structures. Therefore, the use should be critically questioned and should represent an individual case decision. In addition, the performance of BURP can be impeded to the point of impossible by the cervical collar itself. However, the benefit of using BUPR in combination with VL is potential optimization of the procedure based on the fact that the BURP applying person can reassess the effect directly on the VL screen.

A multicenter randomized controlled study compared six different VLs, one of which was C-MAC PM® D-Blade®. The difficult airway was imitated in 720 patients with a cervical collar. In this study, McGrath™ MAC reached a 98% first-attempt success rate followed by C-MAC PM® with a 95% first-attempt success rate. It must be mentioned that only McGrath™ MAC was used with a Macintosh-type blade all participants were familiar with. All other five VLs were attached to highly angulated blades [28]. However, in this study VLs were not tested against each other in the same patient. Despite these restrictions, C-MAC PM® with D-Blade® allowed intubation of 95% of all patients on the first attempt, showing a high rate of successful intubation cases in a simulated difficult airway, which aligns with our findings [29].

Six intubation cases were rated with 0 points, 2 of which were determined CL score I and 4 CL score IIa. Therefore, improvements in CL view were not associated with relevant clinical improvements. Again, the explanation could be the hyperangulated form of the blade. This shape results in a good view of the glottis structures but makes it difficult to maneuver the tip of the tube in the direction of view.

Our investigation has several limitations including small sample size and the impossibility to blind the investigators. A bias can therefore not completely be ruled out. Another limitation was that visualization instead of intubation success was considered as the outcome criterion. Therefore, a link between visualization of the glottic structures and intubation success using three different approaches in one patient could only be investigated using the indirect approach with D-Blade. However due to ethical considerations made by the local ethical committee it was on the one hand necessary to keep the total time of the three laryngoscopies as short as possible and on the other hand to avoid multiple intubation instances due to an increased risk of an intubation associated trauma. Therefore, to reduce the risk for all participants we decided to perform intubation with all devices only by one investigator.

Our study is, to the best of our knowledge, the first that investigates three laryngoscopies in one patient, which has been evaluated before only in manikins. It must be taken into account that most mannequins do not accurately reflect human airway anatomy as is necessary for airway management training [30].

## 5. Conclusion

In this study of human patients, placed in cervical collars to simulate difficult airways in whom a difficult view was obtained, changing from direct laryngoscopy with C-MAC PM® and a Macintosh blade to indirect laryngoscopy with C-MAC PM® and a Macintosh or D-Blade® resulted in a significantly improved view of the glottis structures which can be further improved by using the BURP maneuver.

These findings make D-Blade® in combination with C-MAC PM® a helpful tool in managing difficult airways in emergency cases. Regular training to gain a high level of experience with the used devices seems very important. Nevertheless, there are cases of failed intubation despite good view due to the highly curved shape of D-Blade®. The C-MAC Guide or other similar preformed devices may lead to further success because they follow the curvature of D-Blade® and should be the subject of further investigation.

## Figures and Tables

**Table 1:** Demographic data.

	Age (years)	Height (cm)	Weight (kg)	Collar circumference (cm)
Median	53.5	169.5	73.0	37.5
25/75 percentile	39.5/66.8	163.3/178.0	67.0/84.5	34.0/40.8

Data displayed as median and 25/75 percentile.

**Table 2:** Prognostic factors for difficult ETI.

	Without Stifneck	With Stifneck	*p* value
MS	2 (1/3)	4 (3/4)	< .001
IID (cm)	4.85 ± 1.0	2.50 ± 0.9	< .001
Rec. (°)	47.5 (36.3/60.0)	10.0 (5.0/17.5)	< .001

MS = Mallampati score.

IID = interincisor distance (cm) .

Rec = reclination (°).

Level of significance *p* < .05.

MS + Rec analyzed by Wilcoxon's signed rank test, values shown as median and 25/75 percentile.

IID analyzed by the paired *t*-test, values shown as mean ± SD.

**Table 3:** Improvement of ETI.

	No BURP			BURP			No BURP → BURP				
	DM→VM	DM→VD	VM→VD	DM→VM	DM→VD	VM→VD	DM→DM	DM→VM	VM→VM	VD→VD	DM→VD
No improvement	26	6	5	18	1	0	32	18	17	1	1

Improvement	6	26	13	7	28	15	0	14	1	4	31

*p* value	0.01	<0.001	<0.001	<0.001	<0.001	<0.001	0.433	<0.001	0.0657	0.111	<0.001

Description	Improvement: changing from direct to indirect laryngoscopy with a Macintosh blade without BURP	Improvement: changing from direct to indirect laryngoscopy and a Macintosh to D-Blade® without BURP	Improvement: changing from a Macintosh to D-Blade® in indirect laryngoscopy without BURP	Improvement: changing from direct to indirect laryngoscopy with a Macintosh blade with BURP	Improvement: changing from direct to indirect laryngoscopy and a Macintosh to D-Blade® with BURP	Improvement: changing from a Macintosh to D-Blade® in indirect laryngoscopy with BURP	No Improvement: using additional BURP when performing direct laryngoscopy with a Macintosh blade	Improvement: changing from direct to indirect laryngoscopy with a Macintosh blade with BURP	No Improvement: using additional BURP when performing indirect laryngoscopy with a Macintosh blade	No Improvement: using additional BURP when performing indirect laryngoscopy with D-Blade®	Improvement: changing from direct to indirect laryngoscopy and a Macintosh to D-Blade® with BURP

Since only changes based on Cormack-Lehane grades 3 and 4 were examined and not changes based on grades 2a, 2b, and 1, this table shows differently evaluated numbers of analyzed patients.

Data dichotomized into improvement or no improvement when changing technique or blade.

*p* value calculated using Fisher's exact test. Level of significance *p* < .05.

Improvement = change from poor to good view.

No improvement = persistent poor view.

DM = direct laryngoscopy Macintosh blade.

VM = videolaryngoscopy Macintosh blade.

VD = videolaryngoscopy D-Blade.

**Table 4:** Subjective rating of ETI with D-Blade and C-MAC PM®.

	Grade A	Grade B	Grade C	Grade D
Number (n)	22	10	6	10
Percentage (%)	45.8	20.8	12.5	20.8

Number of ETI rating with D-Blade.

## Data Availability

The data used to support the findings of this study are available from the corresponding author upon request.

## Additional Points

*Article Summary*. (1) Why is this topic important? Managing unexpected difficult airways remains extremely challenging for emergency physicians and occurs in up to 14.8% in emergency situations. Particularly in emergency situations patients receive immobilization of the cervical spine leading to worsening intubation conditions. (2) What does this study attempt to show? This study compares indirect videolaryngoscopy with C-MAC PM® D-Blade® and a C-MAC PM® Macintosh blade with direct laryngoscopy with a C-MAC PM® Macintosh blade regarding visualization of the glottic structures in patients with a simulated difficult airway. All three laryngoscopies were performed in one patient by one investigator. (3) What are the key findings? Using a C-MAC PM® laryngoscope in combination with D-Blade® yielded improved glottic views compared with C-MAC PM® in combination with a Macintosh blade used with either the direct or indirect technique. (4) How is patient care impacted? Videolaryngoscopy has the potential to improve airway management in patients with cervical spine immobilization by obtaining an optimized view of the glottis structures.
